# Follicular dendritic cell sarcoma: a great mimicker with unpredictable clinical course—experience from a tertiary care cancer center in India

**DOI:** 10.3389/fonc.2025.1544803

**Published:** 2025-06-10

**Authors:** Ajas Ibrahim, Mohmad Hussain Mir, Farhana Siraj Bagdadi, Mushtaq Ahmad Sofi, Abrar Rasool Khanday, Suhail Ahmad Wani, Sunil K. Regmi, Mudasir Hamid Bhat, Nisar Ahmad Syed, Faisal R. Guru, Ulfat Ara Wani, Sumyra Khurshid Qadri

**Affiliations:** ^1^ Department of Medical Oncology, Sher-I-Kashmir Institute of Medical Sciences, Srinagar, India; ^2^ Department of General Medicine, Sher-I-Kashmir Institute of Medical Sciences, Srinagar, India; ^3^ Department of Radiation Oncology, Sher-I-Kashmir Institute of Medical Sciences, Srinagar, India; ^4^ Department of Radiodiagnosis and Imaging, Sher-I-Kashmir Institute of Medical Sciences, Srinagar, India; ^5^ Department of Pathology, Sher-I-Kashmir Institute of Medical Sciences, Srinagar, India

**Keywords:** dendritic cells, follicular dendritic cell sarcoma, mimicker, histopathology, immunohistochemistry

## Abstract

**Background:**

Follicular dendritic cell sarcoma (FDCS) is a rare mesenchymal malignant tumor derived from follicular dendritic cells. FDCS arises mainly from lymph nodes and rarely are extranodal. Diagnostic dilemma occurs due to the same micromorphology as other sarcomas and lymphomas. Curative radical resection is the standard therapy, and adjuvant treatment is not defined. For unresectable disease, chemotherapy and radiotherapy are indicated with variable response rates. Due to its rarity, a standard treatment is not yet defined.

**Objective:**

This study aims to analyze the clinicopathological features, treatment patterns, and survival outcome of FDCS cases in our institution.

**Methodology:**

The study was conducted in the Department of Medical Oncology State Cancer Institute, Sher I Kashmir Institute of Medical Sciences (SKIMS), Srinagar, Jammu and Kashmir, India. Biopsy-proven FDCS patients were identified through the hospital-based registry from January 1, 2020 to December 31, 2023.

**Results:**

A total of six patients were diagnosed during the study period. The median age was 28 years (range, 21–51 years). There were four male and two female patients, with male-to-female ratio of 2:1. Common symptoms were abdominal pain (50%) and cough and dyspnea (33.3%). Four patients (66.6%) had nodal involvement with retroperitoneum and mediastinum in two cases each. Three patients had extranodal involvement, with the colon in two and with the liver in one. Five patients were initially misdiagnosed as non-Hodgkin’s lymphoma, soft tissue sarcoma, neurogenic tumor, and carcinoma. The treatments offered were surgery, chemotherapy, targeted therapy, radiotherapy, and observation. Four patients were alive at a median follow-up of 12 months, with three patients having no evidence of disease and one case living with the disease. Two patients had succumbed to the disease.

**Conclusion:**

The study described the clinicopathological characteristics, diagnostic challenges, and management difficulties in FDCS patients. Due to the rarity of this disease, high expertise is needed to diagnose FDCS; otherwise, the diagnosis usually gets delayed.

## Introduction

Follicular dendritic cell ѕаrcоmа (FDCS) is a rare and slowly growing tumor that was first recognized by Monda and Rosai in 1986 (1). It originates from mesenchymal dendritic cells (2). Dendritic cells are immune cells that specialize in antigen presentation and endocytosis, found primarily in peripheral lymphoid organs (3). They are classified into three major subtypes: follicular dendritic cells (FDCs), interdigitating dendritic cells (IDCs), and fibroblastic reticular cells (FRCs). FDCs play a central role in primary and secondary lymphoid follicles by presenting antigens to B cells. Both FDCs and FRCs are derived from mesenchymal tissue, while IDCs originate from hematopoietic cells (4).

FDCS is classified as a type of sarcoma with only a few hundred cases reported to date, with an incidence rate of <0.4% (5). This typically affects young adults in their fifth decade and is rare in children, with no sex predilection (6). FDCS commonly present as a slow-growing localized tumor and rarely as a widespread disease (7). It involves nodal and extranodal sites with varying frequencies, and in some cases, it has mixed involvement (4, 8–11). The commonly involved nodal sites are the neck, mediastinum, axilla, and abdomen, while the liver, lung, and spleen are common extranodal sites (4, 7).

Diagnosing FDCS is challenging due to its histopathological similarities to various other conditions, including non-Hodgkin lymphoma, sarcoma, melanoma, undifferentiated carcinomas, various inflammatory conditions like inflammatory myofibroblastic tumor (IMT), reactive follicular hyperplasia, and other dendritic and histiocytic cell disorders (8, 12, 13). Morphologically, FDCS is characterized by spindled to ovoid cells that form fascicles, whorls, diffuse sheets, or nodules, often with lymphoplasmacytic infiltration. Tumor cells generally express markers associated with follicular dendritic cell differentiation, such as CD21, CD23, and CD35 (14–16).

Due to its rarity, there are no established treatment protocols. Radical surgical resection is the primary treatment for localized disease, while adjuvant radiotherapy has not shown a significant impact on survival (6, 11). Chemotherapy is recommended for unresectable or widespread disease, though an optimal regimen has yet to be identified (6, 16, 17). To date, several hundred cases have been reported worldwide. Here we present our experience of six (6) FDCS patients diagnosed and treated at our oncology center.

## Materials and methods

This retrospective study was conducted in the Department of Medical Oncology, State Cancer Institute at Sher I Kashmir Institute of Medical Sciences (SKIMS), Deemed Medical University, Srinagar, Jammu and Kashmir, India. All biopsy-proven FDCS patients were identified through the hospital-based cancer registry over a 4-year period from January 1, 2020 to December 31, 2023. A total of six cases were diagnosed during the study period. The details about demographics, clinical presentation, radiology, histopathology, treatment offered, and outcome were collected from the patients’ case record files from the Medical Record Section of the State Cancer Institute, SKIMS, and was documented on a designed proforma. Immunohistochemistry was performed in all of the cases on formalin-fixed paraffin-embedded tissues by using a fully automated machine (Ventana Benchmark XT) using monoclonal antibodies to CD117 (Cellmarque−1:100, YR145), CD68 (Cellmarque−1:50 PG−M1), CD34 (DAKO−1:50, QBEnd10), CD45 (Thermo−1:100, RA/RO), CD43 (DAKO−1:50, DF−T1), Desmin (BioGenex−1:30, D33), multiple myeloma 1 (MUM1) (Cellmarque−1:100, MRQ43), CD2 (Cellmarque−1:100, MRQ11), CD3 (DAKO−1:50 F7.2.38), CD10 (Ventana−RTU, SP67), CD23 (Cellmarque−1:50, MRQ12), CD21 (Cellmarque−1:50, MRQ32), CD20 (Cellmarque−1:100, L26), CD79a (Cellmarque−1:100, JCB117), CD1a (BioGenex−1:30, O10), epithelial membrane antigen (EMA) (Cellmarque−1:100, E29), CD19 (Cellmarque−1:50, MRQ36), synaptophysin (Thermo−1:50, SP11), chromogranin A (Cellmarque−1:100, LKH110), human melanoma black−45 (HMB 45) (Cellmarque−1:50, HMB45), and anaplastic lymphoma kinase−1 (ALK−1) (Thermo−1:50, 5A4). Descriptive statistics were analyzed by using the appropriate statistical software SPSS V24. Ethical clearance was granted by IEC SKIMS.

## Results

### Clinical features

**Figure 1:**
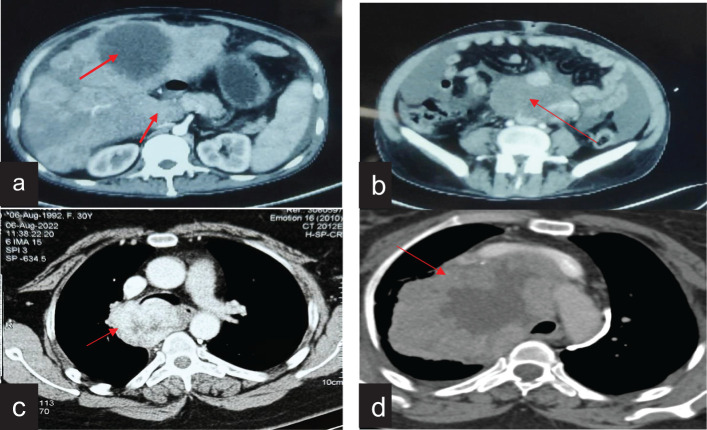
Radiological features of FDCS cases on contrast-enhanced computed tomography. **(a)** CT of the abdomen showing retroperitoneal FDCS with liver metastasis. **(b)** CT of the abdomen showing a large retroperitoneal FDCS. **(c)** CT of the chest showing mediastenial FDCS extending from the lower paratracheal region and abutting the trachea and esophagus. **(d)** CT of the chest showing a bulky mediastinal FDCS causing bronchial compression and SVC syndrome.

In the present study, six patients of FDCS were analysed, with four male and two female patients, respectively (**Table 1**). The median age was 28 years (range, 21–51 years). The common presenting symptoms included abdominal pain and discomfort, cough, breathlessness, and lower back pain. The sites of involvement were the mediastinum (2/6), retroperitoneum (2/6), and cecum (2/6). One patient with mediastinal FDCS had superior vena cava syndrome (case no. 1), and another had been treated for Hodgkin’s lymphoma 8 years back and had concomitant myasthenia gravis with FDCS (case no. 4). *De novo* metastasis to the liver was present in one patient (case no. 3) with retroperitoneal FDCS (**Figure 1**).

**Table 1:** Clinicopathological characteristics and treatment patterns of FDCS patients in our study.

Case number	Age/sex	Site	Dimensions (cm)	Symptoms	Duration of complaints in months	Metastasis	Nodal	Extra Nodal	Initial diagnosis	Initial treatment	Further treatment	Follow-up
1.	51/M	Mediastinum	11 × 9	Dyspnea, cough and neck swellings	1	No	Yes	No	DLBCL	6 cycles R-CHOP followed by RT	Gemcitabine and docetaxel 4 cycles followed by resection and 2 more cycles of the same chemotherapy	Alive
2	24/F	Retroperitoneum	7 × 6	Abdominal pain	4	No	Yes	No	Neurogenic tumor	R0 resection	Nil	Alive
3	29/M	Retroperitoneum	10 × 8	Lower back pain	5	Liver	Yes	Yes	FDCS	Tab. pazopanib 400 BD for 1 year	Gemcitabine and docetaxel × 2 cycles	Died
4	34/F	Mediastinum	6 × 5	Dyspnea and cough	4	No	Yes	No	Pleomorphic sarcoma	Denied surgery and chemotherapy	No	Alive
5	21/M	Cecum	7 × 5	Pain abdomen	2	No	No	Yes	Pleomorphic sarcoma	Right hemicolectomy	6 cycles of gemcitabine and docetaxel	Alive
6	40/M	Cecum	4 × 5	Pain abdomen	3	No	No	Yes	Adenocarcinoma	Right hemicolectomy	No	Died

### Pathologic features

**Figure 2:**
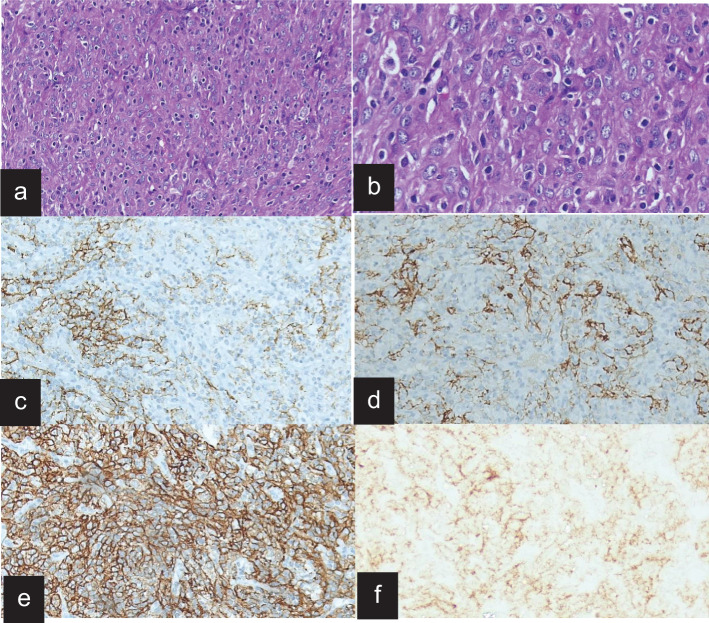
Histopathology and immunohistochemistry of FDCS. **(a)** H&E stain on low power, showing fascicle whorls of spindle cells in a fibro−collagenious stroma. **(b)** H&E stain on high power shows prominent giant cells and mitotic figures and scattered small lymphocytes. **(c)** The tumor cells are immunoreactive for CD21. **(d)** The tumor cells are immunoreactive for CD35. **(e)** The tumor cells are immunoreactive for D2-40 diffusely. **(f)** The tumor cells are immune negative for EMA.

On gross examination, the tumor was well circumscribed and soft to firm with necrosis, and the hemorrhagic areas had sizes ranging from 6 to 11 cm. Microscopically, the tumor was composed of round to spindle cells arranged in sheets in a fascicular and whorled pattern. Lymphoid cells were interspersed in one case and also had giant cells and mitotic figures. On immunohistochemistry, the tumor cells demonstrated positive staining for the follicular dendritic cell markers CD21 (100%), CD23 (100%), and CD35 (50%). Vimentin and D2–40 were done in one patient, who was immunoreactive (**Figure 2**).

### Diagnostic dilemma

Only one patient in our study had upfront diagnosis as FDCS. The rest of the patients (5/6) had an initial diagnosis as either diffuse large B cell lymphoma (1/5), pleomorphic sarcoma (2/5), neurogenic tumor (1/5), or poorly differentiated adenocarcinoma (1/5).

### Treatment and outcome

In three patients (two colon cases and one retroperitoneum case), upfront surgery was done (case numbers 2, 5, and 6). Among the two colonic FDCS, one case received 6 cycles of adjuvant chemotherapy. The patient who was initially diagnosed as DLBCL received 6 cycles of R-CHOP and local radiotherapy, but due to non-responsive disease, he was re-evaluated for an alternative diagnosis. The differentials in such case could be lymphoblastic lymphoma, Hodgkin’s lymphoma, thymoma, small cell carcinoma, extra-gonadal germ cell tumor, etc. A repeat biopsy along with a review of the initial biopsy confirmed FDCS. His mediastinal mass was unresectable. He was started on combination chemotherapy with gemcitabine and docetaxel. After 4 cycles, the reassessment showed a significant reduction in the tumor. He was subjected to R0 resection followed by two more cycles of the same chemotherapy. One patient with retroperitoneal FDCS had liver metastasis at presentation. He received oral pazopanib 400 mg BD for 1 year and upon progression was started on gemcitabine and docetaxel but died after 2 cycles due to disease progression. The patient who had been treated 8 years back for Hodgkin’s lymphoma and now had mediastinal FDCS denied any treatment and opted for observation. One patient in our study received radiotherapy. Two patients (2/6) died due to disease progression. Four patients (4/6) are alive at the time of the last follow-up.

## Discussion

Follicular dendritic cell sarcoma (FDCS) is a very rare mesenchymal tumor of follicular dendritic cell origin (1). FDCS has been a diagnostic challenge since its description as most of the cases are initially labeled as lymphoma, sarcoma, or carcinoma. There is limited literature on this entity. Most of the literature on FDCS are from case reports and case series. A pooled analysis of 462 patients by Saygin C et al. documented a median age of 50 years (4). In contrast, our patient group was generally younger, with a median age of 28 years (ranging from 21 to 51 years). Two-thirds of the patients had abdominal symptoms, which is consistent with other studies on FDCS (4, 15). A diagnostic challenge was encountered in five patients, with initial misdiagnosis including non-Hodgkin’s lymphoma, pleomorphic sarcoma, thymic carcinoma, and poorly differentiated adenocarcinoma. Similar diagnostic discrepancies have been reported in other studies, with rates as high as 58% (4, 10, 18). Immunohistochemistry was the primary method used for confirmation, with all patients (6/6) testing positive for CD21, CD23, and CD35. Typically, follicular dendritic cell (FDC) markers, such as CD21, CD23, CD35, D2-40, clusterin, CXCL13, FDC-secreted protein, and serglycin, are positive in FDCS. In contrast, cytokeratin, CD1a, langerin, lysozyme, and myeloperoxidase stains are negative. Markers like EMA, S100, and CD68 may show variable staining patterns. However, it is not uncommon for some markers to be lost in FDCS cases (7, 19).

Interestingly, “one” patient was treated for Hodgkin’s lymphoma and presented with a mediastinal mass and myasthenia gravis. Gounder M et al. reported that 35% patients had solid tumors, and 3% were reported as lymphoma previously. Their paper suggested that immunosuppression may be a risk factor for FDCS (6). This probability of immunosuppression as a risk factor might be valid as our patient was on prolonged chemotherapy followed by hematopoietic stem cell transplant. The inflammatory variant of follicular dendritic cell sarcoma is aggressive and presents in the liver and spleen. If surgically not amenable, the patients with this variant are more likely to receive systemic therapy (20).

Most of our patients had a localized disease, in line with other studies showing that 85% of FDCS patients present with localized tumors (3). However, despite being localized, the majority of our cases were locally advanced. The patients who underwent surgery remain disease-free, while the others, except for two, are living with the disease. According to a pooled analysis, the 2-year survival rates for early-stage, locally advanced, and metastatic disease are 82.4%, 80%, and 42.8%, respectively (4). In our series, the locally advanced patients also demonstrated good 1-year survival rates, though they experienced significant morbidities. The low proliferation index, indicating a slower tumor growth rate, may explain this outcome. Previous studies have shown that adjuvant chemotherapy or radiotherapy does not improve survival, so no adjuvant treatment was generally recommended for the patients who underwent surgery (4).

The systemic therapies used in various case series and case reports are CHOP, EPOCH, ICE, CVP, gemictabine and docetaxol, methyprednisolone, various VEGF inhibitors, and TKIs with different response rates. The systemic therapies administered to our patients included R-CHOP, gemcitabine and docetaxel, and pazopanib. One patient, initially diagnosed with DLBCL, was treated with R-CHOP and radiotherapy but showed no improvement. Two other patients received gemcitabine and docetaxel upon disease progression. In a large, single-center study by Jain et al., the overall response rate to gemcitabine and docetaxel was 80%, while the CHOP regimen resulted in only a partial response in 60% (21). In our study, the patient who received pazopanib experienced a stable disease for 1 year but eventually progressed. Preclinical studies suggest that vascular endothelial growth factor (VEGF) inhibits the maturation and activation of dendritic cells, which could explain why pazopanib, with its anti-VEGFR properties, was effective. FDCS is also linked to BRAF mutations, EGFR overexpression, PDL1/2 overexpression, and potential involvement of the mTOR pathway. Radiotherapy is primarily used for palliation of symptoms. Targeted agents against these pathways could be explored in clinical trials (6).

### Strengths and weaknesses

FDCS is a rare condition with diagnostic and therapeutic challenges. This study has tried to highlight the diagnostic dilemma and challenges in treating these patients. However, the sample size of this study is small in view of the rarity of the condition.

## Conclusion

Our study described the clinicopathological characteristics and management challenges in FDCS. Due to the rarity of this disease, high expertise is needed for the diagnosis of FDCS; otherwise, it will lead to a delay in diagnosis. For resectable disease, surgery remains the standard of care. The role of the adjuvant and the choice of chemotherapy are challenging with only limited results, and radiotherapy is used in palliative settings only. Further research into the pathogenesis and management of FDCS is required for better outcomes of this rare entity.

## Data Availability

The raw data supporting the conclusions of this article will be made available by the authors, without undue reservation.
